# Effects of body position on autonomic regulation of cardiovascular function in young, healthy adults

**DOI:** 10.1186/1746-1340-15-19

**Published:** 2007-11-28

**Authors:** Nobuhiro Watanabe, John Reece, Barbara I Polus

**Affiliations:** 1 Clinical Neuroscience Research Group, Division of Chiropractic, School of Health Sciences, RMIT University, Melbourne, Australia; 2 Division of Psychology, School of Health Sciences, RMIT University, Melbourne, Australia

## Abstract

**Background:**

Analysis of rhythmic patterns embedded within beat-to-beat variations in heart rate (heart rate variability) is a tool used to assess the balance of cardiac autonomic nervous activity and may be predictive for prognosis of some medical conditions, such as myocardial infarction. It has also been used to evaluate the impact of manipulative therapeutics and body position on autonomic regulation of the cardiovascular system. However, few have compared cardiac autonomic activity in supine and prone positions, postures commonly assumed by patients in manual therapy. We intend to redress this deficiency.

**Methods:**

Heart rate, heart rate variability, and beat-to-beat blood pressure were measured in young, healthy non-smokers, during prone, supine, and sitting postures and with breathing paced at 0.25 Hz. Data were recorded for 5 minutes in each posture: Day 1 – prone and supine; Day 2 – prone and sitting. Paired *t*-tests or Wilcoxon signed-rank tests were used to evaluate posture-related differences in blood pressure, heart rate, and heart rate variability.

**Results:**

Prone versus supine: blood pressure and heart rate were significantly higher in the prone posture (*p *< 0.001). Prone versus sitting: blood pressure was higher and heart rate was lower in the prone posture (*p *< 0.05) and significant differences were found in some components of heart rate variability.

**Conclusion:**

Cardiac autonomic activity was not measurably different in prone and supine postures, but heart rate and blood pressure were. Although heart rate variability parameters indicated sympathetic dominance during sitting (supporting work of others), blood pressure was higher in the prone posture. These differences should be considered when autonomic regulation of cardiovascular function is studied in different postures.

## Background

As body requirements change, autonomic output regulates cardiac function (e.g., heart rate), to maintain a stable internal environment [1]. At first glance, the heart appears to beat regularly, however, the interval between one heartbeat and the next is not the same. Further, embedded within these beat-to-beat variations in length of interval between successive heartbeats are inherent rhythms, at specific frequencies. These changing frequencies constitute what are referred to, collectively, as heart rate variability (HRV) and can be revealed through power spectral analysis. The power spectrum characterises the strength (or power) of these frequencies and reflects sympathetic and parasympathetic (vagal) contributions to their generation. That is, a power spectral analysis of HRV can be used, non-invasively, to quantify sympathetic and parasympathetic output to the heart.

Decreased HRV may be predictive of poor prognosis of myocardial infarction and cardiac failure [2] and has been used to evaluate the impact of manual therapeutic procedures (such as spinal manipulation and massage) on cardiac autonomic nervous activity. Budgell and co-workers examined effects of manipulation on HRV in young, healthy adults: upper cervical spine (patients supine) [3] and thoracic spine (patients prone) [4]. Delaney *et al.*[5] compared HRV parameters before and after trigger point therapy to the head, neck, and back (patients sitting). McNamara *et al.*[6] examined modulation of sympathetic and parasympathetic components of autonomic drive to the heart (before cardiac catheterisation), during back massage of patients in the lateral recumbent posture.

Body position significantly influences cardiac autonomic drive in humans. In healthy adults, heart rate variability has been compared across supine and right- and left-side lying postures [7]; supine and right-side lying postures [8]; supine and sitting postures [9]; supine and standing postures [10-12]; and supine, standing, and head-up and -down tilt postures [13]. In healthy adults, autonomic balance does not change significantly with different recumbent postures [7,8], but is clearly different between supine and vertical postures (standing or sitting). Sympathetic nervous function predominates in vertical postures, while vagal function predominates in recumbent postures.

Autonomic function also has been examined in patients who were prone and under general or spinal anaesthesia [14], and in chronic heart failure patients in right and left side-lying and supine postures [15,16]. In patients with heart disease, the right recumbent posture is associated with enhanced vagus activity (when compared with supine and left recumbent postures) [15,16].

To date, there have been no direct comparisons of cardiac autonomic output in supine and prone postures, or in prone and sitting postures. Although commonly assumed by patients undergoing manual therapy, effects of these postures on autonomic and cardiovascular function may differ.

We sought to establish the impact of recumbent and sitting postures on autonomic regulation of cardiovascular function. Other factors can modify cardiovascular adjustments to changes in posture. For example, elderly people with systolic hypertension show poor cardiovascular adjustments to changes in posture from horizontal to vertical when compared with normotensive elderly people [17]. Because we focused on healthy, young adults, this particular modifying factor was presumed absent.

## Methods

Our study was approved by the RMIT Human Research Ethics Committee. Written, informed consent was obtained from participants before commencement of experiments, and all study protocols were conducted in accordance with the Declaration of Helsinki.

Eligible participants were between 18 and 35 years old and in good general health. Nineteen young adults responded to advertisements placed around the RMIT University campus, but four were excluded: due to high blood pressure (two), medication use (one), and benign arrhythmia (one). Participants (nine males and six females) were 24 ± 3 years old and had a body mass index of 22.2 ± 3.5 kg/m^2 ^(expressed as mean ± standard deviation [SD]). None were smokers, used medication, or had a history of cardiovascular disease, diabetes mellitus, or cancer. Prior to the experiment, to assess general health status and account for factors that might influence autonomic and cardiovascular activity, participants completed general health, cardiovascular, and pre-experimental questionnaires. These focused on medical history, current health status, tobacco and medication use, and food and caffeine intake. Participants also completed questionnaires after each experimental session, regarding unpleasant sensations or discomforts during the experiment. Discomfort was assessed using a 10 cm, visual analogue scale (VAS), where 0 indicated "complete comfort" and 10 "worst pain imaginable."

### Measurement of autonomic function

Heart rate (HR), HRV, and systolic and diastolic blood pressure (BP) were measured. A 3-lead electrocardiogram (ECG) allowed measurement of quick changes in HR [18], and visualisation of the QRS waveform. Disposable electrodes (Blue Sensor, Medicostest, Denmark) were positioned, with the negative electrode over the manubrium and the positive and earth electrodes at the left and right axillary lines (over the 5th intercostal space). Signals were amplified (BIO Amp ML 132, ADInstruments, Castle Hill, NSW, Australia) and stored on a personal computer. R-R intervals were calculated (Chart for Windows V 5.1.1 with HRV extension V 1.0.1, ADInstruments, Castle Hill, NSW, Australia) and the power spectrum of HRV was derived for the period of each intervention. The high frequency (HF) (0.15-0.4 Hz) component of the HRV power spectrum reflects parasympathetic activity [19] and the low frequency (LF) (0.04–0.15 Hz) reflects a combination of sympathetic and parasympathetic activity [19]. The ratio of LF to HF (LF/HF) was adopted to determine the predominance of cardiac sympathetic nervous activity. We did not calculate the power of the very low frequency component (0–0.04 Hz), because it is unreliable over short recording periods [19].

### Measurement of cardiovascular function

A Portapres^® ^(Model-2, Finapres Medical Systems, The Netherlands) continuously measured BP and HR, using a finger cuff around the middle finger of the right hand. The Portapres^® ^uses a hydrostatic height correction to transform measured BP values to those expected at the level of the heart (cf. [20]). Results were transferred to the data acquisition system (Chart for Windows) and displayed on a computer monitor, in real time.

### Posture definition

Autonomic and cardiovascular functions were measured during prone, supine, and sitting postures. Participants were encouraged to position themselves comfortably, but once settled, were asked to remain still for recording.

#### Prone

Participants laid horizontally on a treatment table with hands on hand rests. The headrest was designed to facilitate participants' breathing and was adjusted to minimise neck flexion, extension, and rotation.

#### Supine

Participants lay on the table with a contoured pillow supporting their natural cervical lordosis.

#### Sitting

A custom-designed chair supported participants' upright posture while minimising body and head movement. Footrests permitted comfortable knee flexion and both seat cushion and back support were provided. Immediately prior to recording, a helmet frame fixed the participant's head in a neutral position.

### Experimental procedures

Recordings of HR and BP in the three postures were made in an air-conditioned laboratory, with white noise minimising disturbing sounds. Participants were asked to abstain from food and caffeine-containing beverages for at least 4 hours prior to data collection, and from alcoholic beverages and exercise for at least 12 hours. Two experiments (prone versus supine and prone versus sitting) were conducted on different days. To help minimise diurnal variation, participants were encouraged to schedule each experiment for the same time of day.

The vestibular system is responsible for balance [21], and is thought to influence autonomic and cardiovascular activities [22-24]. Therefore, to minimise vestibular organ activation, participants were instructed to avoid head motion during recording; they were also encouraged to stay awake. Adjustment of autonomic function to a particular posture is thought to occur within 5 minutes [18]. Therefore, to stabilise autonomic outflow to cardiovascular organs before definitive recordings for each posture, participants were asked to make themselves more comfortable, and then remain still for 5 minutes. Additionally, through respiratory sinus arrhythmia, a participant's respiratory rhythm can influence HRV components [25]. To standardise this impact, participants were asked (following the rest period) to synchronise their breathing to a metronome set at 0.25 Hz (15 times a minute) for 5 minutes.

#### Day 1: prone-supine

In the prone posture, HR and BP were measured continuously during both resting and breath-synchronised phases. Participants then moved to a supine posture, and recordings were repeated.

#### Day 2: prone-sitting

Identical to Day 1, except that prone posture was followed by sitting posture.

To confirm normal autonomic nervous function [26], at the end of Day 2, participants were asked to place a hand in a bucket of icy water (the cold pressor test), for as long as they could tolerate, but not longer than 1 minute. Blood pressure and HR were monitored during the test; although not required, had a significant sudden drop in BP and/or HR been observed, we would have terminated the procedure and excluded the participant from the study. Participants were also excluded if they did not respond to this test.

### Data analysis

We recorded HR, mean arterial pressure, and systolic and diastolic BP, during rest and synchronised breathing periods in each posture. Mean values of each parameter were computed using Chart for Windows and analysed with the statistical software package SPSS (V 12.0.1 for Windows, SPSS Inc., U.S.A).

Electrocardiographic data recorded during synchronised breathing periods in each posture were analysed off-line (Chart V 5.1.1 with HRV extension for Windows V 1.0.1) for frequency spectrum characteristics, including LF and HF (absolute and normalised), and LF/HF. Paired samples *t*-tests were used to compare postures. When measurements for a variable deviated markedly from the normal distribution, the Wilcoxon signed-rank test was used (and reported as z scores). The statistical significance level for each comparison was set at *p *< 0.05.

Reproducibility of cardiovascular and autonomic parameters on different days was examined via the intraclass correlation coefficient (ICC). Values above 0.75 were considered to indicate good reliability, lower values poor to moderate [27]. Paired samples *t*-tests were conducted to check for consistent differences in these parameters across recording days.

To evaluate cold pressor test response, minimum values of BP and HR were compared against mean values of BP and HR recorded during synchronised breathing periods in the sitting posture. A normal response to the cold pressor test was defined as a change in BP and/or HR to at least 2 standard deviations of reference values.

## Results

A few participants reported a small degree of experiment-related discomfort (VAS = 0.64 ± 1.69 on Day 1, 0.12 ± 0.52 on Day 2). All responded normally to the cold pressor test.

### Day 1: prone-supine

**Figure 1:**
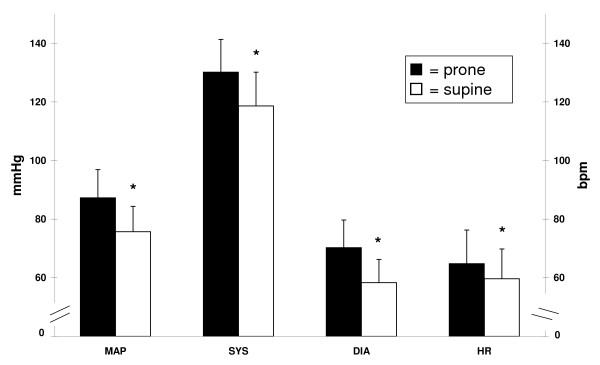
**Comparison of blood pressure and heart rate between two horizontal postures (prone ▪ and supine ▫)**. Expressed as mean ± SD and compared using paired *t*-tests. Left scale applies to MAP, SYS, and DIA, right scale only to HR; DIA = diastolic blood pressure, HR = heart rate, MAP = mean arterial pressure, SYS = systolic blood pressure, BPM = beats per minute, and * = statistically significant difference from value in prone posture.

Differences in HRV components between prone and supine postures are presented in Table 1, and mean arterial pressure, systolic and diastolic BP, and HR are shown in Figure 1. Between prone and supine postures, mean arterial pressure [*t *(14) = 6.28, *p *< 0.001, *d *= 1.26], systolic BP [*t *(14) = 4.56, *p *< 0.001, *d *= 1.01], diastolic BP [*t *(14) = 7.26, *p *< 0.001, *d *= 1.38], and HR [*t *(14) = 5.04, *p *< 0.001, *d *= 0.48] were all significantly higher in the prone posture than in the supine. In contrast, components of HRV did not differ between postures: total power (TP) [*z *(15)*= *-0.74, *p *= 0.46], LF [*z *(15) = -1.53, *p *= 0.13], normalised LF [*t *(14) = -0.042, *p *= 0.97, *d *= 0.01] HF [*z *(15) = -1.53, *p *= 0.26], normalised HF [*t *(14) = -0.13, *p *= 0.90, *d *= 0.03], and LF/HF [*t *(14) = 0.24, *p *= 0.81, *d *= 0.07].

**Table 1:** Comparison of heart rate variability parameters on Day 1 and Day 2; N = 15.

	**Day 1**			**Day 2**		
	
	**Prone**	**Supine**	**(*p*)**	**Prone**	**Sitting**	**(*p*)**
**TP (ms^2^)**	4896.39 ± 5579.34	4076.27 ± 4215.74	0.46 (w)	5004.72 ± 5797.60	2746.36 ± 2643.12	0.26 (w)
**LF (ms^2^)**	1023.57 ± 1505.68	963.55 ± 1095.13	0.13 (w)	800.09 ± 791.30	667.95 ± 731.74	0.61 (w)
**LF (nu)**	40.70 ± 21.12	40.93 ± 19.85	0.97 (t)	37.61 ± 18.46	59.87 ± 20.88	0.001 (t)
**HF (ms^2^)**	2069.20 ± 3292.70	1975.40 ± 3304.52	0.26 (w)	2145.69 ± 3320.78	577.78 ± 650.45	0.001 (w)
**HF (nu)**	55.91 ± 21.22	56.61 ± 19.67	0.90 (t)	58.42 ± 18.12	35.84 ± 20.87	< 0.001 (t)
**LF/HF**	0.98 ± 0.76	0.93 ± 0.69	0.81 (t)	0.82 ± 0.65	3.00 ± 3.07	0.001 (w)

Expressed as mean ± SD; (*t*) = paired *t*-test, (w) = Wilcoxon signed-rank test, HF = high frequency of HRV power spectrum, LF = low frequency, LF/HF = ratio of low frequency to high, TP = total power, ms^2 ^= milliseconds squared, nu = normalised unit.

### Day 2: prone-sitting

**Figure 2:**
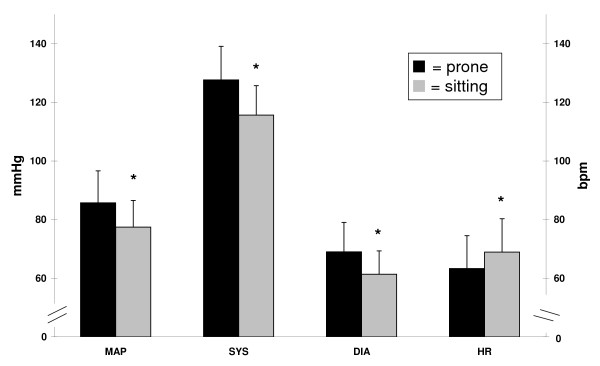
**Comparison of BP and HR between the horizontal (prone ▪) and vertical (sitting ) postures**. Expressed as mean ± SD and compared using the paired *t*-tests. Left scale applies to MAP, SYS, and DIA, right scale only to HR; DIA = diastolic blood pressure, HR = heart rate, MAP = mean arterial pressure, SYS = systolic blood pressure, BPM = beats per minute, and * = statistically significant difference from a value in the prone posture.

Differences in HRV components between prone and sitting postures are presented in Table 1, and BP and HR in Figure 2. Both autonomic and cardiovascular parameters differed between postures. In the prone posture, mean arterial pressure [*t *(14) = 5.32, *p *< 0.001, *d *= 0.96], systolic BP [*t *(14) = 5.84, *p *< 0.001, *d *= 1.36], and diastolic BP [*t *(14) = 5.73, *p *< 0.001, *d *= 1.01] were significantly higher, and HR [*t *(14) = -3.61, *p *= 0.003, *d *= 0.55] was significantly lower. For HRV during sitting, normalised LF values were significantly higher [*t *(14) = 4.38, *p *= 0.001, *d *= 1.13] and both normalised and absolute HF values were significantly lower [*t *(14) = 4.76, *p *< 0.001, *d *= 1.16] and [*z *(15)*= *-3.18, *p *= 0.001]. These differences were reflected in LF/HF, which was also significantly higher in the sitting posture [*z *(15) = -3.35, *p *= 0.001]. Between postures, TP [*z *(15)*= *1.14, *p *= 0.26] and absolute LF [*z *(15) = -0.51, *p *= 0.61] were not significantly different.

### Reproducibility of autonomic nervous and cardiovascular parameters

Parameters measured in the prone posture on days 1 and 2 were used for reproducibility analysis. Table 2 presents descriptive data and ICC values for all HRV components. Table 3 shows reproducibility of BP and HR values. Several components of HRV (TP, normalised LF, and absolute and normalised HF) and HR demonstrated good reliability. Cardiovascular parameters, including mean arterial pressure and BP (systolic and diastolic), showed poor reproducibility.

**Table 2:** Reproducibility of heart rate variability parameters recorded in the prone posture on two different days; N = 15.

	**ICC**
**TP (ms^2^)**	0.95*
**LF (ms^2^)**	0.74
**LF (nu)**	0.78*
**HF (ms^2^)**	0.95*
**HF (nu)**	0.78*
**LF/HF**	0.69

ICC = Intraclass correlation coefficient, HF = high frequency of HRV power spectrum, LF = low frequency, LF/HF = ratio of low frequency to high, TP = total power, ms^2 ^= milliseconds squared, nu = normalised unit, * = good reproducibility (ICC > 0.75).

**Table 3:** Reproducibility of blood pressure and heart rate recorded in prone posture on two different days; N = 15.

	**Prone Day 1**	**Prone Day 2**	**ICC**
**MAP (mmHg)**	87 ± 9.7	86 ± 9.8	0.13
**SYS (mmHg)**	130 ± 11.2	129 ± 9.8	0.25
**DIA (mmHg)**	70 ± 9.4	70 ± 8.7	0.062
**HR (bpm)**	65 ± 11.5	63 ± 11.4	0.86*

Expressed as mean ± SD; ICC = Intraclass correlation coefficient, DIA = diastolic blood pressure, HR = heart rate, MAP = mean arterial pressure, SYS = systolic blood pressure, bpm = beats per minute, * = good reproducibility (ICC > 0.75).

## Discussion

We recorded HR, HRV, and beat-to-beat BP, as measures of autonomic and cardiovascular function, comparing prone and supine postures (Day 1) and prone and sitting postures (Day 2). We also examined reproducibility of these parameters, measured in the prone posture on both days.

Parameters of autonomic (HRV) and cardiovascular (BP and HR) activity were affected less by changes between the two horizontal postures (prone and supine) than changes between horizontal (prone) and vertical (sitting). Between prone and supine, there was no significant difference in HRV parameters indicative of a change in autonomic balance to the heart, but HR and BP were significantly higher in the prone posture. In contrast, between prone and sitting postures, there were significant differences in the balance of autonomic drive to the heart, with a shift towards sympathetic dominance during sitting.

Others have examined cardiovascular regulation during different horizontal postures in adults. For example, Pump *et al.*[28] observed cardiovascular parameters over a period of 9 hours: supine posture for 3 hours, then either supine or prone for 6 hours. In the prone posture, HR, total peripheral resistance, and sympathetic nerve activity increased, and stroke volume decreased. However, there was no difference in BP between these two postures. Tabara *et al.*'s [29] study design was similar to ours. Cardiovascular variables were measured over a short time frame, with participants in the supine and then prone postures. Unlike our study, these authors found that BP in the prone posture was significantly lower than in the supine. Changes in HR reported by Tabara et al. were similar to ours and those reported by Pump *et al.*[28].

In contrast to our results, Pump et al. observed no change in BP between supine and prone postures. Tabara et al. did observe a change, but BP was lower in prone than supine. These discrepancies probably resulted from differences in methodology: Tabara et al. measured cardiovascular variables only 1 minute after posture changed from supine to prone and Pump et al. recorded parameters at the start and every 90 minutes thereafter, for 9 hours. After a shift from supine to standing, 1 to 2 minutes may be required to stabilise consequent cardiovascular adjustments and up to 5 minutes to complete most autonomic adjustments [18]. Cardiovascular function is controlled by regulatory mechanisms involving the neural, renal, and endocrine systems, each operating within a different time frame. For example, baro- and chemoreceptors are involved in cardiovascular adjustments as soon as arterial pressure is altered, whereas blood volume control by the kidneys plays a role in blood pressure regulation several hours later [30]. To minimise the impact that the very act of changing postures might have on our results, we had participants maintain each posture for 5 minutes prior to data collection. Therefore, the different changes observed between Pump *et al.*'s [28] or Tabara et al.'s study [29] and ours may be because each study recorded cardiovascular activity at a different phase of the cardiovascular adjustment cycle. The mean age of volunteers for Tabara *et al.*[29] was 50 ± 11 years, for us it was 24 ± 3 years. Finally, approximately one-third of Tabara et al.'s participants were considered hypertensive (although they had no history of cardiovascular disease and were not being treated for hypertension). We employed only healthy volunteers, with no signs or symptoms of hypertension.

We showed significantly higher HR and BP in the prone posture than the supine. Toyota and Amaki [31] measured haemodynamic changes associated with prone posture during general anaesthesia in surgical patients and observed decreases in end-systolic and end-diastolic left ventricular area and left ventricular volume. They ascribed these changes to reduction of venous flow (caused by compression of the inferior vena cava) and augmentation of left ventricular filling resistance (caused by compression of the thorax) during the prone posture [31]. This has been supported by Pump *et al.*[28], who postulated that compression of the thorax might have been responsible for their observed decreased stroke volume. In turn, this was thought to attenuate arterial pulse waves, which inhibited baroreflexes and subsequently increased sympathetic nervous activity. Thus, the condition of the cardiovascular system is thought to be quite different during prone and supine postures. A decrease in central blood flow may cause pooling and increased blood volume in peripheral vessels. Consequently, vessel constriction is induced and BP increased (the Bayliss myogenic response [32]). The prone posture is likely to cause facial tissue compression that does not occur during the supine posture, and trigeminal afferents from there modulate cardiac function [33]. Therefore, in movements between prone and supine postures, cardiovascular parameters (HR and BP) might be regulated by different reflexive neural and non-neural factors. An example of a non-neural factor could be vessel myogenic activity, which is not associated with the cardiac autonomic nervous system.

In our study, HRV parameters indicative of autonomic nervous activity to the heart did not differ between the two horizontal postures and were associated with a large standard deviation (reflecting large individual differences within our sample). Further, calculation of Cohen's *d *revealed a very small effect size. Based on these results, post-hoc analysis revealed that approximately 50 participants would have been required to reliably detect (power = 0.8) a difference in HRV parameters between prone and supine postures. With our sample and the effect of posture change on cardiac autonomic activity so small, HRV analysis could not reveal related differences in autonomic regulation of the heart.

With a horizontal to vertical posture change, a hydrostatic gradient is introduced and cardiovascular adjustments may occur to maintain adequate perfusion to the brain. We found that HR increased and HRV parameters indicated a shift to sympathetic dominance during the sitting posture. In contrast, BP was higher in the prone than in the sitting posture. Arterial BP is a product of cardiac output (heart rate × stroke volume) and total peripheral resistance [34]. Shamsuzzaman *et al.*[35] has shown that antigravity muscle activity influences vasomotor and cardiovascular activity during postural change. It is likely that the sitting posture minimised antigravity muscle involvement. Therefore, one possible explanation for our finding that BP was higher in the prone posture than in the sitting, may be that there was little change in total peripheral resistance secondary to vascular compression induced by skeletal muscle contraction, resulting in a minimal change in BP during the sitting posture.

We demonstrated that components of HRV are highly reproducible, across days, which is consistent with former studies [36,37]. Kowalewski and Urban [37] used a 12-month follow-up and found that components of HRV were consistent. Others, however, have asserted that HRV parameters are not a consistent tool for measuring autonomic nervous function [38,39]. There are several possible explanations for this contradiction. First, a minimal 2 to 5 minute recording period is required for accurate HRV analysis [19]. Toyry et al.'s use of a 1-minute period [38] may have made it hard to assess whether measures of HRV are reproducible. For HRV analysis, respiration rate is usually fixed, and because this influences the location of the central frequency within the high frequency band of the power spectrum [40]. Lord et al.'s study [39] set the respiration rate at 0.167 Hz, which may have resulted in inadequate separation of LF and HF components of the power spectrum, leading to decreased reproducibility. It is critical that measures of HRV be conducted under well-controlled circumstances.

Finally, we demonstrated consistency in HR and HRV components across different recording days. Measurements of BP, however, varied from day to day within individuals. Because their comfort was a priority, participants determined their final body position and daily BP measurements may have been influenced by changes in central blood flow, due to different pressures on the vena cava [31]. Another explanation might be diurnal variations in participants' hydration levels. Under normal conditions, plasma osmolality regulates vasopressin secretion [41], which in turn constricts blood vessels [1]. Our participants were asked to forgo food and caffeinated beverages for 4 hours prior to data collection, and alcoholic beverages for 12 hours. Otherwise, food and fluid intake was not governed. A participant's plasma osmolality may have varied daily, and this may have influenced recorded BP.

## Conclusion

Comparing prone and supine postures, cardiac autonomic nervous activity was so variable among participants that we could detect no differences. However, heart rate and mean arterial, systolic, and diastolic blood pressure all were significantly greater in the prone position. Comparing prone and sitting postures, HRV parameters indicated sympathetic dominance during sitting (supporting work of others), and BP was higher during the prone posture. In studies of effects of interventions on autonomic regulation of cardiovascular function, such posture-related difference must be considered.

## Abbreviations

BP Blood pressure

ECG Electrocardiogram

HF High frequency

HR Heart rate

HRV Heart rate variability

ICC Intraclass correlations

LF Low frequency

LF/HF Ratio of low frequency to high frequency

RSA Respiratory sinus arrhythmia

SD Standard deviation

TP Total power

VAS Visual analogue scale

## Competing interests

The author(s) declare that they have no competing interests.

## Authors' contributions

NW performed volunteer recruitment, data collection, and data analysis (of HR, HRV, and BP), and participated in design of the study, statistical analysis, and drafting the manuscript. JR was the statistical consultant and participated in statistical analysis and drafting the manuscript. BIP participated in the design of the study, statistical analysis, and drafting the manuscript. All authors read and approved the final manuscript.
